# Silver Nanoparticles: Synthesis, Detection, Characterization and Assessment in Environment

**DOI:** 10.3390/nano12010167

**Published:** 2022-01-04

**Authors:** Mònica Iglesias

**Affiliations:** Department of Chemistry, University of Girona, C/M. Aurèlia Capmany, 69, 17003 Girona, Spain; monica.iglesias@udg.edu

The number of studies on silver nanoparticles (AgNPs) has risen in recent years due to the increase in their use in different commercial products, the concerns regarding their release in the environment, as well as their toxicological effects. Although nanosilver toxicology is still not completely clear, the hazards it poses for living organisms must be considered.

The aim of this Special Issue, entitled “Silver Nanoparticles: Synthesis, Detection, Characterization and Assessment in Environment”, is to collect current studies in the field of AgNPs synthesis and characterization for different purposes, as well as those related to advanced analytical methodologies and sample treatment procedures designed to improve their determination and understand their behavior, both in the environment and in relation to living beings. This Special Issue comprises eleven research articles and two reviews. Most of the studies use classical AgNPs with different sizes and coatings, but some of them uses more innovative variations of this products, such as silicon-based silver dendritic nanoforest [1] or starch-capped AgNPs [2].

Among the eleven original studies included in this Special Issue, four of them are related to the synthesis and use of AgNPs for different purposes. In the first study, silicon-based silver dendritic nanoforests showed remarkable antibacterial activity against Escherichia coli and Staphylococcus aureus, which are enhanced under light illumination [1]. Another work synthesized starch-capped AgNPs and studied their possible use as cytotoxic agents against prostate cancer cells. The study concluded that starch-capped AgNPs successfully damage cancer cell lines; therefore, they are proposed as anticancer agents [2]. Alumina-supported AgNPs were also proposed as catalysts for betulin oxidation. In this study, AgNPs were deposited on different alumina supports and characterized before its use as catalysts. The study found that their catalytic behavior depends on the support nature and the preparation and treatment methods used [3]. AgNPs were also used as a surface plasmon resonance colorimetric sensor for Ni^2+^ determination. This study includes the synthesis of AgNPs functionalized with mercaptoundecanoic acid and their capability to detect micromolar levels of nickel ions in the presence of other common metal cations [4].

Three of the works presented in this Special Issue are related to toxicological effects. The first one studies the effect of AgNPs on cell viability in human lung fibroblasts and concludes that AgNPs of 10 nm and 75 nm induced immunomodulatory responses and reduced metabolic activities in this type of cell [5]. A second toxicological study evaluated the phytotoxicity of AgNPs on tobacco plants [6]. This study compares AgNPs with different coatings and indicates that citrate has less severe effects in terms of photosynthesis of tobacco plants than polyvinylpyrrolidone and cetyltrimethylammonium bromide ones; the authors link these findings with the stability of the AgNPs. The last toxicological study focusses on rat liver damage produced by silver nanorods with golden cores. Although the authors found significant changes in toxicological parameters in rats exposed to the silver nanorods, after 6 weeks, the changes mostly returned to the normal levels [7].

Other three studies are related to the analysis and characterization of AgNPs in different matrices. The first one characterized 14 different commercial products that supposedly contain AgNPs. Authors found important variability between production batches of the same product, significant differences between measured and labelled silver concentrations and different types of silver forms present in the products [8]. Another study explores the stability of citrated capped AgNPs using asymmetrical-flow and field-flow fractionation. The different aggregation behaviors of AgNPs in several environmental water matrices were observed. The aggregation rate of AgNPs seems to be related with ionic strength, dissolved organic matter and halides concentration in waters [9]. The separation of AgNPs and ionic silver in different types of water samples using a cation exchange resin is also studied. The separation enables the determination of AgNPs in the presence of ionic silver by inductively coupled plasma optic emission spectroscopy and the accurate particle size determination by single particle inductively coupled plasma mass spectrometry [10].

A study monitoring spray coating of AgNPs on textiles is also included in this Special Issue. The work aims to improve the spray process by optimizing AgNPs deposition and reducing either wastes and workers exposure [11].

As indicated, two reviews are also included in this Special Issue. The first summarizes the impacts of the release of AgNPs in the environment, their toxicological effects on living beings (mainly plants and soil microorganisms), and the possibilities of phytoremediation. The review concludes that more research is still needed to improve the strategies to remove AgNPs from the environment [12].

A review of the efficiency and mechanisms of different AgNPs and their composites as antibacterial agents ends this Special Issue. The possibilities but also the toxicological effects of these nanomaterials are discussed in order to take advantage of their benefits but to avoid their risks [13].

To sum up, I would like to thank the authors of all the studies for their excellent work, and the reviewers for their constructive contributions. Finally, a special thanks to the editorial board for their help during all the process of setting up this Special Issue. I hope all readers will enjoy it.

## References

[1] Huang H.J., Chang H.-W., Lin Y.-W., Chuang S.-Y., Lin Y.-S., Shiao M.-H. (2020). Silicon-Based Ag Dendritic Nanoforests for Light-Assisted Bacterial Inhibition. Nanomaterials.

[2] Morais M., Machado V., Dias F., Palmeira C., Martins G., Fonseca M., Martins C., Teixeira A., Prior J., Medeiros R. (2021). Starch-Capped AgNPs’ as Potential Cytotoxic Agents against Prostate Cancer Cells. Nanomaterials.

[3] Grigoreva A., Kolobova E., Pakrieva E., Mäki-Arvela P., Carabineiro S.A.C., Gorbunova A., Bogdanchikova N., Murzin D.Y., Pestryakov A. (2021). Supported Silver Nanoparticles as Catalysts for Liquid-Phase Betulin Oxidation. Nanomaterials.

[4] Rossi A., Zannotti M., Cuccioloni M., Minicucci M., Petetta L., Angeletti M., Giovannetti R. (2021). Silver Nanoparticle-Based Sensor for the Selective Detection of Nickel Ions. Nanomaterials.

[5] Löfdahl A., Jern A., Flyman S., Kåredal M., Karlsson H.L., Larsson-Callerfelt A.-K. (2020). Silver Nanoparticles Alter Cell Viability Ex Vivo and in Vitro and Induce Proinflammatory Effects in Human Lung Fibroblasts. Nanomaterials.

[6] Štefanić P.P., Košpić K., Lyons D.M., Jurković L., Balen B., Tkalec M. (2021). Phytotoxicity of Silver Nanoparticles on Tobacco Plants: Evaluation of Coating Effects on Photosynthetic Performance and Chloroplast Ultrastructure. Nanomaterials.

[7] Liu Y., Wen H., Wu X., Wu M., Liu L., Wang J., Huo G., Lyu J., Xie L., Dan M. (2021). The Bio-Persistence of Reversible Inflammatory, Histological Changes and Metabolic Profile Alterations in Rat Livers after Silver/Gold Nanorod Administration. Nanomaterials.

[8] De Leersnyder I., Rijckaert H., De Gelder L., Van Driessche I., Vermeir P. (2020). High Variability in Silver Particle Characteristics, Silver Concentrations, and Production Batches of Commercially Available Products Indicates the Need for a More Rigorous Approach. Nanomaterials.

[9] Boughbina-Portolés A., Sanjuan-Navarro L., Moliner-Martínez Y., Campíns-Falcó P. (2021). Study of the Stability of Citrate Capped AgNPs in Several Environmental Water Matrices by Asymmetrical Flow Field Flow Fractionation. Nanomaterials.

[10] Iglesias M., Torrent L. (2021). Silver Nanoparticles and Ionic Silver Separation Using a Cation-Exchange Resin. Variables Affecting Their Separation and Improvements of AgNP Characterization by SP-ICPMS. Nanomaterials.

[11] Trabucco S., Ortelli S., Del Secco B., Zanoni I., Belosi F., Ravegnani F., Nicosia A., Blosi M., Costa A.L. (2021). Monitoring and Optimisation of Ag Nanoparticle Spray-Coating on Textiles. Nanomaterials.

[12] Ihtisham M., Noori A., Yadav S., Sarraf M., Kumari P., Brestic M., Imran M., Jiang F., Yan X., Rastogi A. (2021). Silver Nanoparticle’s Toxicological Effects and Phytoremediation. Nanomaterials.

[13] Wahab A., Li L., Li H., Abdala A. (2021). Silver Nanoparticle-Based Nanocomposites for Combating Infectious Pathogens: Recent Advances and Future Prospects. Nanomaterials.

